# Prediction in the Aging Brain: Merging Cognitive, Neurological, and Evolutionary Perspectives

**DOI:** 10.1093/geronb/gbac062

**Published:** 2022-04-16

**Authors:** Rachel M Brown, Stefan L K Gruijters, Sonja A Kotz

**Affiliations:** Institute of Psychology, RWTH Aachen University, Aachen, Germany; Faculty of Psychology, Open University of the Netherlands, Heerlen, The Netherlands; Faculty of Psychology and Neuroscience, Maastricht University, Maastricht, The Netherlands

**Keywords:** Aging, Evolution, Learning, Predictive processing, Senescence

## Abstract

Although the aging brain is typically characterized by declines in a variety of cognitive functions, there has been growing attention to cognitive functions that may stabilize or improve with age. We integrate evidence from behavioral, computational, and neurological domains under the hypothesis that over the life span the brain becomes more effective at predicting (i.e., utilizing knowledge) compared to learning. Moving beyond mere description of the empirical literature—with the aim of arriving at a deeper understanding of cognitive aging—we provide potential explanations for a learning-to-prediction shift based on evolutionary models and principles of senescence and plasticity. The proposed explanations explore whether the occurrence of a learning-to-prediction shift can be explained by (changes in) the fitness effects of learning and prediction over the life span. Prediction may optimize (a) the allocation of limited resources across the life span, and/or (b) late-life knowledge transfer (social learning). Alternatively, late-life prediction may reflect a slower decline in prediction compared to learning. By discussing these hypotheses, we aim to provide a foundation for an integrative neurocognitive–evolutionary perspective on aging and to stimulate further theoretical and empirical work.

Aging populations worldwide present an urgent need to understand all sides of cognitive aging: losses, heterogeneity, and gains. Some cognitive capacities clearly suffer with age, at different individual rates (Cabeza et al., 2018; Reuter-Lorenz & Park, 2014). Notable among the vulnerable capacities is the ability to acquire new information, or to learn (Reuter-Lorenz & Park, 2010). At the same time, the capacity to accumulate and to utilize generalized knowledge seems to be stable and may even improve (Salthouse, 2019; Spreng & Turner, 2019). This observed profile requires explanation. Understanding may be gained by merging neurocognitive descriptions of how the brain ages with an evolutionary understanding of why organisms age as they do. In particular, merging these views can address both how, and why, cognitive performance can both decline and improve over the life span—questions that remain challenging for a “deficit” view of aging. We propose that (a) over the life span the human brain becomes more effective at generating predictions relative to learning and that (b) a shift from learning to prediction over the life span may have evolved due to prediction’s adaptive value or its robustness to decline.

Prediction is a neurocognitive construct that broadly refers to inferring the future based on knowledge of the past (Bar, 2007; Bubic et al., 2010; Clark, 2013). The term “prediction” is used to denote a process, synonymous with “anticipation” or “projection,’’ as well as the resulting content, synonymous with “an expectation” (Bubic et al., 2010). The basis for prediction is memory. Prediction exploits the rich associations the human brain acquires and stores over the long term to help make sense of incoming sensory information that is missing, incomplete, coarse, or noisy (Bar, 2007). Prediction involves retrieving long-term memories, comparing memory traces to incoming sensory information, and utilizing those memory traces to infer a current or future state of the world that is uncertain. This process has been described cognitively as one of “analogy” and “association” (Bar, 2007), where sensory information is compared to similar information in memory, which in turn activates additional associations that collectively form a prediction. If a listener cannot hear a word well enough to identify it, she can guess the word based on what it sounded like. She can also guess that the words she will hear next will be related in meaning to the words she has already heard (Obleser & Kotz, 2010). Neurologically, prediction may be related to signal flow from neural networks for long-term associative memory (such as the default-mode network) to networks that more directly receive sensory input from the environment (such as subcortical and primary sensory regions; Bar, 2007; Bubic et al., 2010; Turner & Spreng, 2015). Prediction may improve, or provide a viable alternative to learning, as long-term memories accumulate, and knowledge becomes more extensive and generalizable (abstracted) over the life span (Moran et al., 2014; Spreng, Lockrow, et al., 2018).

Learning and prediction can be seen as separate cognitive processes: in learning, sensory information is used to change long-term memory (Barron et al., 2015; but see de Houwer et al., 2013). Conversely, in prediction, long-term memory is used to interpret sensory information or decrease uncertainty about future states (Bar, 2007). Prediction and learning are necessarily linked: learning changes predictions, and prediction changes learning (Clark, 2013). For instance, if a prediction is inaccurate, detecting this inaccuracy may initiate learning in order to change, and improve, the previous prediction (Clark, 2013). The above definitions of learning and prediction parallel the behavioral *exploration–exploitation* distinction (Hills et al., 2015). Exploration refers to seeking new resources with uncertain outcomes, which can be seen as integral to the learning process (see Spreng & Turner, 2021). Exploitation refers to utilizing existing resources with more certain outcomes (Hills et al., 2015; Spreng & Turner, 2021): prediction can therefore be seen as a process of exploitation (specifically, exploiting knowledge resources). We argue that aging may improve or increase reliance on prediction as learning declines, analogous to a proposed exploration–exploitation shift across the life span (see Gopnik, 2020; also see Spreng & Turner, 2021), and we discuss potential explanatory hypotheses for this shift.

We here review recent empirical work in neurocognitive aging and link this evidence to evolutionary theory in order to assess the hypothesis that aging is not characterized by deterioration alone (Reuter-Lorenz & Park, 2014; Spreng & Turner, 2019). Notably, evolutionary principles are particularly well-suited to provide answers to why-questions about phenomena: in this case, why human-typical aging is marked by specific patterns of cognitive change (e.g., Bateson & Laland, 2013). Applying this interdisciplinary perspective, we first argue that the evidence to date tentatively suggests that aging brains become effective at predicting (i.e., exploiting long-term memories) compared to learning. We then offer several explanations which explicitly draw from evolutionary theory. We end by suggesting avenues for further work and discussing limitations. First, we review evidence for a learning-to-prediction shift at the behavioral, computational, and neurological levels.

## From Learning to Utilizing Acquired Knowledge: A Neurocognitive Hypothesis

### Cognitive Changes: Increased Reliance on Previously Acquired Knowledge

A common thread in cognitive aging appears to be the increasing difficulty with acquiring novel information from the environment. Cognitive aging is typically associated with decline in episodic encoding—that is, acquiring memories for events that include details about the context in which they were embedded (Naveh-Benjamin et al., 2003). Older adults show impairment relative to younger adults when encoding novel associations, such as combinations of items or the context in which information is presented, but they show less impairment when remembering individual items (which should be familiar; Chalfonte & Johnson, 1996; de Chastelaine et al., 2016; Naveh-Benjamin et al., 2003; Old & Naveh-Benjamin, 2008). Older adults also have trouble relearning or “unlearning” recently acquired associations that are no longer relevant. This type of interference, termed “proactive interference,” has been associated with aging in a variety of tasks, and it is at least partially distinguishable from aging effects on processing speed, working memory, and inhibition (Friedman & Miyake, 2004; Matamales et al., 2016; Pettigrew & Martin, 2014).

In contrast to deficits in acquiring and updating memories, older adults have shown stable or improved performance in tasks that utilize long-term memories. Compared to younger adults, older adults demonstrate similar or enhanced performance on tests of semantic memory, sometimes called “crystallized knowledge” (Craik & Bialystok, 2006), which refers to long-term knowledge that is generalized and abstracted from the context in which it was learned (Allen et al., 2002; Luo & Craik, 2008; Nyberg et al., 1996; Spaniol et al., 2006; Spreng, Lockrow, et al., 2018). Large-scale cross-sectional and longitudinal studies of cognitive performance over the life span demonstrate a highly consistent pattern of loss and gain: while episodic memory declines steadily over the life span, crystallized knowledge such as vocabulary increases steadily throughout adulthood, levels off around age 60, and modestly declines around age 70 (Salthouse, 2014, 2019). This knowledge accumulation itself may impair learning, by interfering with novel information. For example, simulated word-pair association learning showed that increasing levels of individual linguistic experience, independent of age, predicted declines in novel associative learning (Ramscar et al., 2017). On the other hand, accumulated knowledge may help older adults learn information that aligns with their existing knowledge, possibly enabling them to continue acquiring knowledge (such as vocabulary) into late life (Salthouse, 2019). For instance, learning new words in one’s native language should be easier than learning a new language. Although older adults showed reductions in word retrieval, they also tended to produce words that were more common and semantically related to each other (Taler et al., 2020). In addition, differences between older and younger adults in episodic memory performance reduce or disappear when new information is supported by familiar semantic information (for a review, see Spreng & Turner, 2019). For instance, older and younger adults remembered pairs of words with similar accuracy when the words were related in meaning or syntax (Badham et al., 2012; Castel, 2005). As a whole, this evidence suggests that older adults utilize their long-term knowledge, particularly semantic knowledge, more effectively than they learn new information. This proposal aligns with the idea of a shift from explorative to exploitative cognitive modes over the life span (Spreng & Turner, 2021). Similarly, we expect aging to increase reliance on strategies that exploit existing knowledge, while decreasing strategies involved in learning (e.g., novelty seeking). Exploitation of long-term knowledge may additionally offer an alternative strategy to compensate for learning declines (Reuter-Lorenz & Park, 2014).

### Computational Changes: Unreliable Sensation and Reliable Knowledge

Older adults may rely on prediction as sensory signals become less reliable. Computational work suggests that memory and sensation are weighted according to their salience and stability (Clark, 2013; Feldman & Friston, 2010; Wolpe et al., 2016). The more salient and stable the sensation, the more likely it will be to override contradictory expectations (Feldman & Friston, 2010). Likewise, highly stable memory traces may persist despite contradictory sensory signals (Clark, 2013). There is ample evidence that sensation becomes less acute with age, presumably due to decline in peripheral sensory organs and reduced sensitivity in the central nervous system (Guerreiro & Van Gerven, 2011; Lin et al., 2011; Pichora-Fuller & Singh, 2006; Voytek et al., 2015). Older adults show reduced cortical electrical responses to stimulus changes and repetitions (Cheng et al., 2013; Kisley et al., 2005; Moran et al., 2014; Ruzzoli et al., 2012), and they adjust their movements less and more slowly in response to sensory feedback (Buch et al., 2003; Seidler, 2006). Deficits in sensation or sensorimotor adaptation could contribute to deficits in encoding or updating information (Bernard & Seidler, 2014; Li & Lindenberger, 2002). Sensory deficits may relate to reliance on prediction. For instance, older adults showed reduced tactile acuity, which correlated with greater attenuation (presumably overprediction) of self-generated tactile feedback (Wolpe et al., 2016). Sensory impairments may contribute to learning deficits and/or a greater reliance on prediction.

**Figure 1.**
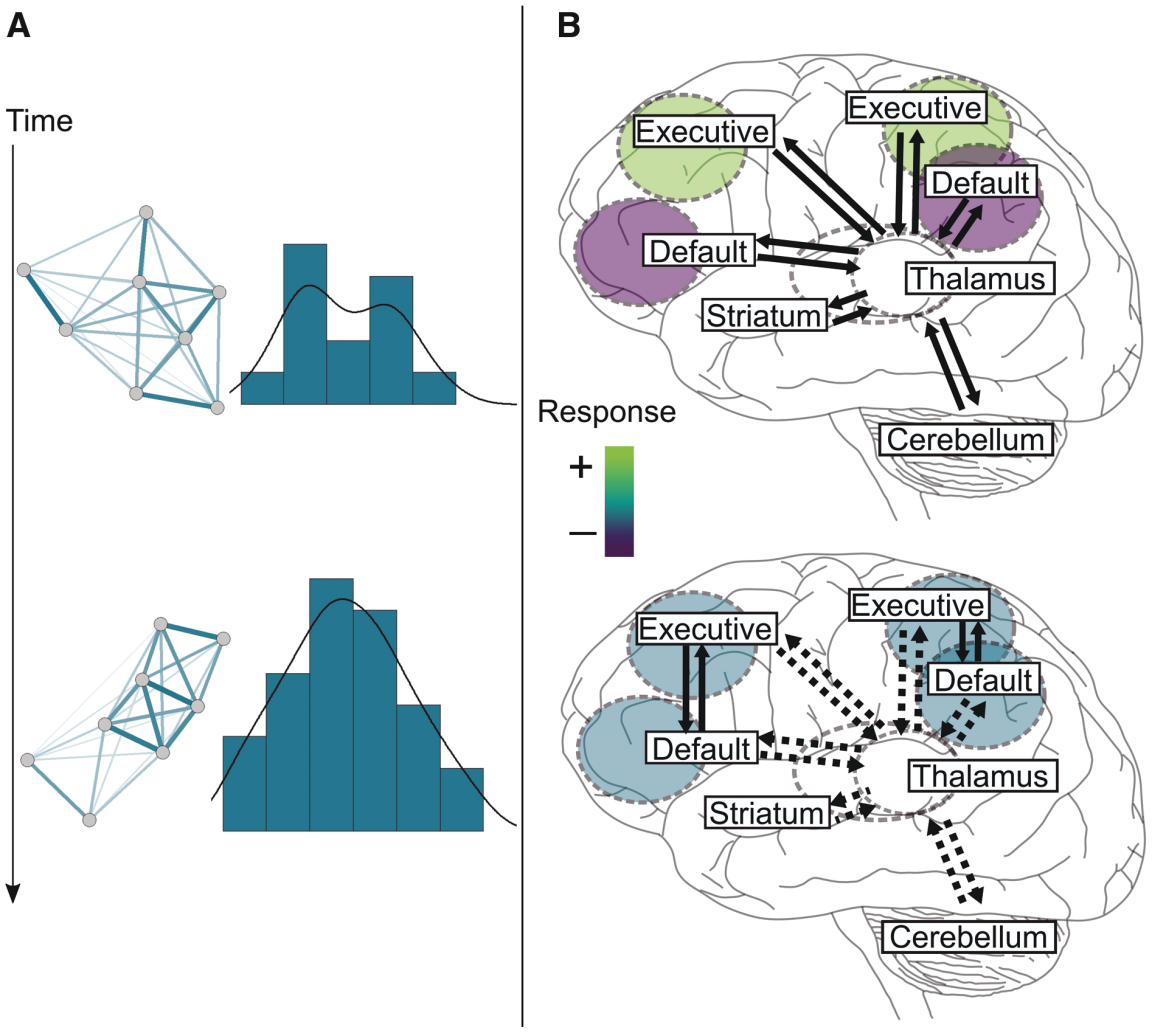
Improved predictive capacity and increased access to memory traces in aging. (A) This panel illustrates hypothesized knowledge stability and abstraction increases over the life span. Stability is illustrated as the strength of input/output connections in a hypothetical network. Over time, higher connection weights (thicker lines) among nodes (dots) result in fewer but highly efficient activation patterns. Abstraction is illustrated as a hypothetical knowledge distribution that acquires a “simpler” unimodal shape (Moran et al., 2014). (B) This panel illustrates hypothesized neural changes which may contribute to increased utilization of knowledge over the life span: subcortical–cortical communication decreases and default-executive coupling (synchrony) increases (Spreng & Turner, 2019). In the upper brain, the lighter color (“+”) indicates increased activation in the executive network, and the darker color (“−”) indicates decreased activation in the default network, during a task. In the lower brain, the color in between light and dark (between “+” and “−”) indicates reduced task modulation in the executive and default networks. Solid arrows indicate increased connectivity, and dashed arrows indicate decreased connectivity. Full color version is available within the online issue.

Older adults may also rely on prediction as their memory traces undergo both quantitative and qualitative changes over time. Quantitatively, memory traces should become more extensive and cover a wider range of information over the life span, as suggested by increases in crystallized knowledge from early adulthood to middle age (Salthouse, 2014). This quantitative change may improve prediction accuracy. Qualitatively, memory traces may grow more stable and generalizable with repeated retrieval and consolidation (Figure 1A), which should enable prediction efficiency. A similar idea is that memories become abstracted over time as they lose contextual detail (“semanticization”), and these abstracted memories may be more efficiently retrieved than contextualized (episodic) memories (Spreng, Lockrow, et al., 2018; Spreng & Turner, 2019). Abstracted knowledge can also be described computationally as a “simpler” predictive model of the world, which, compared to more precise or contextualized models, should enable efficient prediction by accommodating a wide range of information in a variety of contexts (Moran et al., 2014; Figure 1A). Over time, being able to generate accurate and efficient predictions with a given knowledge base should further increase reliance on that knowledge, because knowledge successfully utilized is likely to be maintained and reused (Clark, 2013). In late life, prediction may improve, or become a useful alternative to learning, as more extensive knowledge can be utilized in a generalizable way.

### Neural Systems Changes: Altered Subcortical–Cortical Networks

Studies are now showing that aging changes the functional organization of large-scale neural networks (Zonneveld et al., 2019), as evidenced, for instance, by the tendency of older adults to engage different or additional networks to perform the same tasks as younger adults (Cabeza, 2002; Park & Reuter-Lorenz, 2009; Reuter-Lorenz & Cappell, 2008; Reuter-Lorenz & Park, 2014). A reorganization that may underlie a learning-to-prediction shift is captured by the Default-Executive Coupling Hypothesis of Aging (DECHA; Turner & Spreng, 2015). This hypothesis proposes that aging increases the interaction between a cognitive control, or executive control, network (“executive network”) comprising lateral frontal–parietal regions (Cole et al., 2013) and the default-mode network (“default network”). The default network includes lateral-temporal and medial frontal–temporal–parietal regions, including the hippocampus (Buckner et al., 2008), and it is thought to be involved in memory retrieval (e.g., retrieving learned associations between objects or concepts) and memory-driven processes such as self-reflection and imagining the future (Buckner et al., 2008; Raichle, 2015; Spreng, Madore, et al., 2018; Spreng & Turner, 2019). Thus, it is also conceptualized as a network for prediction (Bar, 2007). Younger adults typically show greater executive network activity and less default network activity while focused on a task (Andrews-Hanna et al., 2014), and this anticorrelation increases as tasks become more difficult (e.g., increased working memory load; Kennedy et al., 2017; Rieck et al., 2017). Compared to younger adults, older adults show less disengagement of the default network, and more default-executive synchrony, when performing difficult tasks (Grady et al., 2016; Turner & Spreng, 2015). Older adults also show less modification of executive network activity as task difficulty changes (Kennedy et al., 2015, 2017; for a review, see Spreng & Turner, 2019; see Figure 1B). The DECHA hypothesis suggests that default-executive synchrony reflects increased utilization of acquired knowledge to guide goal-directed behavior, and it may reflect an exploration-to-exploitation shift over the life span (Spreng & Turner, 2019, 2021). Similarly, default-executive synchrony could reflect a learning-to-prediction shift. Networks involved in guiding goal-directed behavior (executive network) may increasingly rely on generalizable knowledge (via the default network) to solve current tasks, compared to learning new information. In line with this idea, greater spontaneous default-executive synchrony in older adults correlated with a greater proportion of semantic compared to episodic content in autobiographical recall (Spreng, Lockrow, et al., 2018).

Working from this network-reorganization hypothesis, it may be informative to additionally consider the cerebellum and basal ganglia (multiple nuclei including the striatum), given their well-established roles in age-vulnerable cognitive capacities, including associative learning, updating previously learned associations, and cognitive control (Bernard & Seidler, 2014; Bostan & Strick, 2018; Caligiore et al., 2017; Matamales et al., 2016; Schwartze & Kotz, 2013). These subcortical regions are densely connected to the cerebral cortex via reciprocal subcortico–thalamo–cortical loops (for reviews, see Bostan & Strick, 2018; Caligiore et al., 2017), and they show connectivity with default and executive networks, among others (Bernard et al., 2012; Gordon et al., 2021). Transneuronal tracing in nonhumans and human neuroimaging also show disynaptic pathways between the cerebellum and basal ganglia (Bostan & Strick, 2018; Milardi et al., 2016; Pelzer et al., 2013). It is also apparent that these nodes, along with their cortical and mutual connections, are vulnerable to aging. Human neuroimaging shows gray and white matter volume reduction in these regions as a function of age, paralleling cortical volume declines (Barrick et al., 2010; Bernard et al., 2015; Crivello et al., 2014; Fjell et al., 2013; Gellersen et al., 2021; Han et al., 2020; Raz et al., 2005; Resnick et al., 2003; Tamnes et al., 2013). In addition, spontaneous (resting state) subcortico–cortico connectivity reduces with age, including cerebellar–cortical (Bernard et al., 2013, 2021; Ferreira et al., 2016), and striatal–prefrontal (Bo et al., 2014; Su et al., 2018) connectivity. Striatal– prefrontal white matter shows age-related microstructure declines (Samanez-Larkin et al., 2012; Vik et al., 2015; Webb et al., 2020; Ystad et al., 2011), which correlate with reduced executive function performance (Webb et al., 2020), and with reduced cue–reward association learning with age (Samanez-Larkin et al., 2012). Finally, cerebellar–striatal resting state connectivity has been shown to reduce with age (Bernard et al., 2013, 2021; Bo et al., 2014; Hausman et al., 2020) and to correlate with working memory performance (Hausman et al., 2020).

Given these cerebellar–striatal–cortical declines, we speculate that there may be a potential link between reduced cerebellar–striatal–cortical communication (Bernard et al., 2013; Hausman et al., 2020; Webb et al., 2020) and increased default-executive synchrony (Spreng & Turner, 2019). An emerging concept of an integrated cerebellar–striatal system is that it adjusts and updates cortical routines on complementary timescales: the basal ganglia determines relevant goals through reinforcement learning, and the cerebellum determines how to attain those goals through error correction and fine-tuning (Bostan & Strick, 2018; Caligiore et al., 2017). In this view, the cerebellar–striatal system is crucial for optimally updating cortical processes at coarse (striatal) and fine-grained (cerebellar) levels, based on new information (“tutoring” the cortex; Caligiore et al., 2017, p. 210). If this model is applied to the aging brain, it could be further hypothesized that the executive network relies less on cerebellar–striatal updating (e.g., learning) and more on the default network’s knowledge resources enabling prediction (see Figure 1B). This change may occur as (a) novel sensory inputs become less frequent, salient, or relevant, and/or (b) generalizable knowledge resources (a range of cortical repertoires and routines) provide sufficient flexibility to solve current tasks without sensory-guided adjustments (Spreng & Turner, 2019). In line with this idea, a meta-analysis suggested that older adults may underrecruit the cerebellum in working memory tasks compared to younger adults (Bernard et al., 2020). Alternatively (or in parallel), reduced cognitive control over the life span (Spreng & Turner, 2021), notably associated with declining striatal–frontal networks (Buckner, 2004), may be a determining factor in a learning-to-prediction shift. We speculate that an underused cerebellar–striatal system may contribute to declines in learning and a shift toward prediction.

## Learning or Utilizing Acquired Knowledge: Evolutionary Perspectives

Based on the observed cognitive, computational, and neurological changes that occur during healthy aging, we argue that the aging brain shifts from learning to prediction. The aging brain may become more adept at exploiting the outcomes of previous learning. Drawing from evolutionary principles, we offer two adaptive explanations and one “by-product” explanation for a life-span learning-to-prediction shift. The two proposed adaptive explanations explore whether the occurrence of a learning-to-prediction shift can be explained by (changes in) the fitness effects of learning and prediction over the life span. The first hypothesis is that prediction may optimize the allocation of limited resources across the life span. The second hypothesis is that prediction may have prolonged positive fitness effects by contributing to social learning. Lastly, according to a “by-product” hypothesis, a learning-to-prediction shift may be explained as a mere by-product of the mechanisms of human senescence. We briefly explore each hypothesis.

### Adaptationist View: Optimal Cognitive Aging Under Resource Constraints

Evolutionary theory predicts that traits and activities are favored by selection insofar as they contribute to fitness. We define fitness as the combined direct and indirect effects of traits and activities on germline survival (reproductive success), known as inclusive fitness (Gardner & West, 2014). As a general principle, to be a fitness-maximizing organism requires putting sufficient resources into three life-span activities: growing (e.g., developing physiological and psychological functions), maintaining the soma (e.g., repair and immune functions), and reproduction (e.g., mating, gestation, and parenting). Life history theory (Del Giudice et al., 2016; Hill, 1993; Nettle & Frankenhuis, 2020; Stearns, 2000) describes optimal allocation of limited resources to the various fitness-relevant activities of organisms. A critical premise in the life history framework is that no organism can invest unboundedly in growth, maintenance, and reproductive efforts (Hill, 1993; Kirkwood & Rose, 1991). To reproduce, organisms need first to grow and then maintain a functional soma, which requires resources such as time, effort, and energy. Ubiquitous external sources of mortality (e.g., cumulative likelihood of illness, accidents, predation over time) provide the key constraint to do so on a strategic time schedule (Williams, 1957). Biological systems thus need to allocate finite resources (e.g., time and energy) to traits in ways that maximize fitness over the life span (Kirkwood & Rose, 1991). Investing time and energetic resources in one task (e.g., a protracted development of the brain) comes at the expense of other activities (e.g., age of first reproduction). Overall, life history theorists assume that natural selection favors biological systems that strategically allocate their limited resources to development (i.e., growth), somatic maintenance, and reproductive activities (Stearns, 1989, 2000).

These ideas are also foundational to the disposable soma theory of senescence (Kirkwood, 1977; Kirkwood & Rose, 1991). Senescence occurs in most sexually reproducing life-forms. Disposable soma theory suggests that because organisms have a limited resource pool, perfect and indefinite somatic maintenance comes at too large an expense to reproductive activities (Kirkwood, 1977; Kirkwood & Austad, 2000; Kirkwood & Rose, 1991). Or conversely, reproductive functions consume resources that would be needed for indefinite somatic maintenance and repair. Senescence then, rather than an inevitable negative consequence of being, is viewed as a by-product of natural selection prioritizing reproduction over longevity of the soma (Kirkwood & Rose, 1991; Stearns, 2000; Williams, 1957). In sum, life history theory and the disposable soma theory imply that—all else being equal—costly growth and maintenance activities will be kept at a pragmatic minimum by natural selection, depending on their contribution to overall fitness. Learning can be seen as one such costly “growth” activity (Del Giudice et al., 2016; Gopnik, 2020).

For organisms to “grow” by learning they need to be able to adjust their internal (e.g., neural and cognitive) structures in response to input from the environment—that is, learning requires phenotypic plasticity. *Phenotypic plasticity* can be broadly defined as “the degree to which cues received during development affect an organism’s phenotype” (Fawcett & Frankenhuis, 2015, p. 1; see also DeWitt et al., 1998; Van Buskirk & Steiner, 2009). Nearly all species demonstrate phenotypic plasticity, from metamorphosis in insects to changing color pigments in plants. Phenotypic plasticity enables organisms to match their phenotypes to the environment in ways that benefit fitness (DeWitt et al., 1998). Models have shown that the degree of plasticity varies between-species as well as between-individuals, but also within the lifetime of a single individual (Fawcett & Frankenhuis, 2015; Frankenhuis & Walasek, 2020). To understand the changes in plasticity over the life span, it is first important to note that—all else being equal—high degrees of phenotypic plasticity are costly relative to a fixed phenotype (DeWitt et al., 1998; Fawcett & Frankenhuis, 2015; Snell-Rood, 2013). For instance, populations of *Drosophila* flies selectively bred for high learning ability showed reduced reproductive success compared to those bred for low learning ability (Mery & Kawecki, 2003). Further among these costs are the energy demands of cells that can sense cues in the environment and implement the appropriate responses, and the risks incurred by acquiring unreliable information about the environment (DeWitt et al., 1998; Walasek et al., 2021). These factors constrain selective pressure on plasticity (Snell-Rood, 2013). However, the benefits of plasticity may often outweigh its costs. When environmental conditions fluctuate, developing organisms might risk phenotype–environment mismatch. Natural selection might favor plasticity to minimize such mismatch. High levels of plasticity are often favored when organisms have access to reliable cues which convey information about current and future conditions (Fawcett & Frankenhuis, 2015; Frankenhuis & Walasek, 2020; Walasek et al., 2021). In addition, age is itself a factor that can be expected to moderate degrees of plasticity (Fawcett & Frankenhuis, 2015; Walasek et al., 2021). For example, a recent model found that plasticity likely declines across the life span, when the reliability of cues decreases (Walasek et al., 2021). When the reliability of cues increases across some portion of development, plasticity first increases early in development, before decreasing. In addition, the reliability of cues may decrease across the life span because individuals’ sensory systems deteriorate with age, as discussed previously. Under these conditions, individuals may process cues less accurately, further constraining the degree of plasticity.

Overall, given a fixed cost and decreasing fitness benefits of plasticity, the degree of plasticity—and hence learning—is expected to decrease as individuals age (Fawcett & Frankenhuis, 2015; Frankenhuis & Walasek, 2020). Early-life learning is also costly but it supports reproductive fitness throughout the life span (see, e.g., Gopnik, 2020). The motoric, linguistic, and social skills gained in childhood can yield fitness dividends throughout adulthood, including the ability to find reproductive partners, provide resources, and care for offspring. In late life, the brain’s capacity to predict, or utilize knowledge, may become an increasingly cost-effective alternative to learning. These increased benefits of prediction in late life (given fixed costs, decreasing benefits with age, and high initial payoffs of plasticity) may explain why natural selection favors minimal investment in late-life learning, and why it may favor prediction as a cost-effective alternative.

### Adaptationist View: Social Learning

Based on evolutionary theories of postreproductive longevity, another plausible hypothesis is that late-life prediction has adaptive value in the context of social learning. This hypothesis rests on the idea that postreproductive individuals whose traits can no longer influence fitness directly (by contributing to their own reproductive ability) can nonetheless enhance the fitness of kin, and thereby gain an indirect fitness advantage (Gardner & West, 2014). As a social species, human postreproductive longevity may have adaptive value for allocating resources to close kin (hence increasing inclusive fitness), such as caring for the young (e.g., grandmothering; Hawkes et al., 1998; Kirkwood & Austad, 2000), or transferring information via teaching (Gurven et al., 2020). Recent work suggests that teaching behavior in social species may have evolved as a cost- effective strategy for optimal information transfer across generations (Gurven et al., 2020). Late-life (postreproductive) teaching behavior may have maximal adaptive value for conveying complex skills that take years to master. Teaching by older adults maximizes fitness gains for the next generation (for instance, by reducing learning costs for the younger generation), and it minimizes the cumulative costs of teaching behavior within the social group by allocating costs away from reproducing or food-producing individuals (Gurven et al., 2020). Building on this model, we suggest that the fitness benefits of late-life information transfer may create a selective pressure on the ability of the aging brain to maintain and utilize knowledge supporting prediction, despite the potential costs of knowledge maintenance. Thus, while selective pressure on traits is classically thought to decline with age (see discussion below), natural selection may still act upon late-life cognitive abilities, assuming they convey indirect fitness benefits through kin.

### Prediction as a By-Product of Senescence: Prediction Declines More Slowly Than Learning

The adaptationist hypotheses above assume that prediction contributes to overall fitness and hence that the brain’s ability to predict in late life has been directly selected for by natural selection. In contrast, a “by-product” hypothesis assumes that a learning-to-prediction shift has no direct functional significance, but rather may be a by-product of the typical pattern of human senescence. It is possible that prediction is subject to the same declines with age as all biological functions, but merely declines slower compared to learning. This expectation aligns with a foundational principle in evolutionary models of aging (Kirkwood, 1977; Williams, 1957): the fitness benefits of any trait tend to decrease with age, as with each passing year the probability of reproduction decreases. In other words, the older the organism, the less specific activities and capabilities can contribute to its lifetime fitness. As a consequence, selection is mostly contingent on traits’ early-life contribution to fitness. For instance, there is evidence that genes that enhance early-life functions can have deleterious effects in late life but are nevertheless maintained by natural selection—because of the higher weight of early-life benefits versus late-life costs on overall fitness (Kirkwood & Rose, 1991). If prediction declines over the life span, then prediction may only appear to improve relative to learning, simply because it declines at a slower rate. This slower decline could be a by-product of the mechanisms of cognitive decline. For instance, accumulated knowledge may be structured in a way that is more robust to decline (e.g., Dubossarsky et al., 2017), or certain overused knowledge, such as habits, may be retrieved efficiently despite decline.

## Scope, Limitations, and Future Directions

We presented two potential adaptationist hypotheses and a by-product hypothesis which may explain the learning-to-prediction shift, and the required prolonged maintenance of long-term memories and knowledge. Though any (or a combination) of the three hypotheses may explain such a shift, we note that in terms of theoretical parsimony, the by-product hypothesis may face some difficulty. In particular, though different paces of decline could explain why particular cognitive capacities are maintained for longer periods, the hypothesis may be question-begging. Specifically, it requires an additional explanation for why specific cognitive capacities decline at a slower rate compared to others. The nonuniformity of cognitive decline is well-documented (Salthouse, 2019; Spreng & Turner, 2019, 2021), as is the ability of older adults to recruit cognitive and/or neural resources in a compensatory manner (Reuter-Lorenz & Park, 2014). By contrast, the adaptationist hypotheses—which we take to be mutually *inclusive—*both single out specific factors (optimal resource allocation and social learning) which could explain why certain cognitive capacities are maintained or improved in late life. Whereas the resource-optimality hypothesis does particularly well in explaining late-life declines in learning, the social learning hypothesis adds a direct fitness benefit of maintaining a knowledge base for the purpose of information transfer. The resource-optimality hypothesis also aligns with cognitive and computational propositions: updating previously learned information becomes more difficult with age (Pettigrew & Martin, 2014), and the reduced complexity of internal models of the world (abstracted, generalizable knowledge) is assumed to enable more efficient retrieval and hence efficient prediction (Moran et al., 2014; Spreng & Turner, 2019). The specific fitness benefits of prediction proposed by these hypotheses (resource-optimality and social learning) require further research (see below).

This paper embarked on a theory development process (see Borsboom et al., 2021; Haig, 2005), taking arguments for the potential existence of a phenomenon (a learning–prediction shift) as a point of departure. We also developed several explanatory hypotheses which could elucidate such a phenomenon, but it is important to note that we remain nearshore. Critically, rather than having directly generalized the learning-to-prediction shift from specific data sets, a broad literature base was used to substantiate that such a shift may characterize aging. Future research could provide more direct empirical evidence that a learning-to-prediction shift occurs—for instance, by more direct empirical examinations of age-dependent changes in the cognitive and neural underpinnings of learning and prediction. As for the developed explanatory hypotheses, a limitation of the current work is that the proposed hypotheses are merely verbally expressed. Though being useful in the larger process of theory development (Borsboom et al., 2021), verbally expressed theories benefit from formalization in (mathematical) models. Formal models may provide an initial test of feasibility, help explore boundary conditions, and allow more specific predictions to be derived (Muthukrishna & Henrich, 2019; Nettle & Frankenhuis, 2020; Smaldino, 2017; Walasek et al., 2021). For instance, a potential contribution to models of plasticity is an added expectation of decreased environmental uncertainty over the life span based on increasingly better predictive models (knowledge) of the environment. Finally, broader theories of senescence could integrate a focus on explanations for specific cognitive improvements in late life, along with decline.

Building on modeling work, empirical work can then test specific predictions of the adaptationist hypotheses. For instance, the resource-optimal hypothesis assumes that prediction becomes less costly than learning over the life span (in terms of either time, effort, or energetic resources). Further work can test, for instance, whether decreased model complexity translates into metabolic efficiency by reducing the number of distinct neural spiking patterns necessary to encode information (Laughlin, 2001). It will also be important to evaluate, via modeling, whether reduced costs of prediction over the life span also translate into fitness gains. To test the social learning hypothesis, it is particularly important to establish whether prediction can convey indirect fitness benefits, such as through improved teaching or caregiving. Given that the by-product hypothesis is the least parsimonious explanation, and that it is not as straightforward to test, it might be seen as a hypothesis of exclusion, if adaptationist hypotheses prove to be insufficient. Finally, as many of the discussed evolutionary theories and principles (e.g., disposable soma theory, plasticity models) do not uniquely apply to human cognition, but to any species capable of learning, our proposals might extend to other species as well (see, e.g., Fawcett & Frankenhuis, 2015; Walasek et al., 2021).

## Conclusion

In sum, the cognitive, computational, and neurological profile of aging may suggest a shift from learning to prediction. Our interpretation of the reviewed literature is that the aging brain increasingly utilizes (a) acquired knowledge, (b) prediction when faced with reduced sensory reliability, and (c) default-executive network coupling. A learning-to-prediction shift may resolve trade-offs in resource allocation over the life span, minimizing costly learning while exploiting previously acquired knowledge, and/or prediction may enhance indirect fitness, by optimizing information transfer to the next generation. Alternatively, a learning-to-prediction shift may reflect a slower decline in prediction, resulting from a lack of selective pressure on late-life cognitive traits and a robustness to decline. Aligning neurocognitive and evolutionary theories of aging offers a comprehensive understanding of both losses and gains. We hope our theoretical suggestions may spark novel inquiries into cognitive aging, not only from a “deficit-perspective,” but also from a “shift-perspective.”
